# Cytotoxic Property of *Grias neuberthii* Extract on Human Colon Cancer Cells: A Crucial Role of Autophagy

**DOI:** 10.1155/2020/1565306

**Published:** 2020-04-01

**Authors:** Luis M. Guamán-Ortiz, Juan C. Romero-Benavides, Alirica I. Suarez, Stephania Torres-Aguilar, Paola Castillo-Veintimilla, Jimmy Samaniego-Romero, Kevin Ortiz-Diaz, Natalia Bailon-Moscoso

**Affiliations:** ^1^Departamento de Ciencias de la Salud, Universidad Técnica Particular de Loja, Loja 1101608, Ecuador; ^2^Departamento de Química y Ciencias Exactas, Universidad Técnica Particular de Loja, Loja 1101608, Ecuador; ^3^Facultad de Farmacia, Universidad Central de Venezuela, Caracas 1050, Venezuela; ^4^Programa Nacional para el Abordaje Multidisciplinario de las Parasitosis Desatendidas en el Ecuador PROPAD, Instituto Nacional de Investigaciones en Salud Pública LIP, Guayaquil 3961, Ecuador

## Abstract

Traditional herbal medicine has become an important alternative in the treatment of various cancer types, including colon cancer, which represents one of the main health problems around the world. Therefore, the search for new therapies to counteract this disease is very active. *Grias neuberthii* is an endemic plant located in the Ecuadorian Amazon region, which has been used in traditional medicine for its pharmacological properties, including its ability to inhibit tumor cell growth, although scientific studies are limited. We have analyzed the effect of this plant on two colon carcinoma cell lines, that is, RKO (normal p53) and SW613-B3 (mutated p53) cells. Among several extracts obtained from various parts of *G. neuberthii* plant, we identified the extract with the greatest cytotoxic potential, derived from the stem bark. The cytotoxic effect was similar on both cell lines, thus indicating that it is independent of the status of p53. However, significant differences were observed after the analysis of colony formation, with RKO cells being more sensitive than SW613-B3. No evidence for apoptotic markers was recorded; nevertheless, both cell lines showed signs of autophagy after the treatment, including increased Beclin-1 and LC3-II and decreased p62. Finally, three chemical compounds, possibly responsible for the effect observed in both cell lines, were identified: lupeol (**1**), 3′-O-methyl ellagic acid 4-O-*β*-D-rhamnopyranoside (**2**), and 19-*α*-hydroxy-asiatic acid monoglucoside (**3**).

## 1. Introduction

Colorectal cancer ranks third in terms of incidence, but second in terms of mortality. [1] Colorectal cancer is increasing in Central and South America due to an ongoing transition towards higher levels of human development. [2] Understanding the mechanisms underlying the effect of apoptotic/autophagic regulators should generate new ideas and opportunities for chemotherapeutic intervention and the potential treatment of cancer patients. [3].

Natural products continue to be an important source of leads for new medicines. Historically, natural products from plants and animals have been the source of virtually all medicinal preparations and, more recently, natural products have continued to enter clinical trials or to provide leads for compounds that have entered clinical trials. [4] The production of secondary metabolites is favored by the different microenvironments. Continental Ecuador is a region with the third-highest density of endemic plant species worldwide. It is shown in [5] that *G. neuberthi* (Lecythidaceae) is endemic to Colombia, Ecuador, and Peru. In accordance with the ethnomedical uses reported in various herbaria from Ecuador and bibliographical references, medicinal uses (including antitumor) described for *G. neuberthii* are related to the digestive system. [6, 7] The objective of this work was to study the effect of *G. neuberthii* extracts in human colon tumor cell lines as cytotoxic agents, understanding the mechanism responsible for inducing cell death, and determining the possible secondary metabolites involved. It is important to determine the type of cell death that natural products might be inducing and whether the activation of the p53 plays an important role in the cytotoxic effect. Thus, we have selected two colon cancer cell lines, one with normal p53 and another with mutated p53.

## 2. Materials and Methods

### 2.1. Plant Material

*G. neuberthii* was collected on a farm in Lumbaqui (00°01′46″ Lat. S; 77°10′24″ Long. O, 366 m.a.s.l) Sucumbios Province of Ecuador. A sample specimen (LOJA-49) was deposited in the Herbarium of Universidad Nacional de Loja, Ecuador, and identified by Xavier Cornejo and Zhofre Aguirre.

### 2.2. Preparation Extract

The aerial parts (leaves, stem bark, fruit, and seed) were reduced to fine particles by grinding to a suitable size and then were dried at 30°C for seven days in dryer trays with air flow.

The dried and ground aerial parts of *G. neuberthii* (4045 g) were macerated at room temperature for 72 h in a light-free environment, with hexane, ethyl acetate, and methanol, sequentially, with 5 L of each solvent; the procedure was repeated three times. The extracts were filtered using filter paper; all extracts were concentrated at 50 mbar and 37°C on a rotary evaporator (Buchi R210, Switzerland), and subsequently stored at 4°C and protected from light until further use.

Thin-layer chromatography using aluminum plates coated with silica gel 60 F254 (Merck, Germany) was performed on each extract.

For biological studies, stock solutions (40 mg/mL) were prepared in dimethylsulfoxide (DMSO–Sigma Aldrich, USA) and stored at −20°C until use. The aliquots were diluted to obtain the appropriate concentrations before use.

### 2.3. Phytochemical Screening

Phytochemical screening to test for the presence of secondary metabolites (alkaloids, terpenoids, steroids, flavonoids, tannins, saponins, and quinones) and proteins, carbohydrates, and fats and oils in the extracts was carried out using standard procedures [8].

### 2.4. Characterization and Identification of Secondary Metabolites

Melting points were determined using a Fisher-Johns apparatus. The ^1^H and ^13^C NMR spectra were recorded at 400 MHz and 100 MHz, respectively, on Varian 400 MHz Premium Shielded Equipment (Varian, USA) using tetramethylsilane as an internal reference. CDCl_3_, C_5_D_5_N, and DMSO-d_6_ were used as solvents; chemical shifts were expressed in parts per million (ppm), and coupling constants (*J*) were reported in Hz.

### 2.5. Extraction and Isolation of Compounds

The most active extract, methanol extract of stem bark (GNSbM), was partitioned, and 30 g was dissolved in MeOH : H_2_O (9 : 1) in a ratio of 1 : 20 (extract: solvent) (540 mL of MeOH and 60 mL of H_2_O) and sequentially partitioned three times with 400 mL of each solvent hexane (Hex), dichloromethane (DCM), and ethyl acetate (EtOAc) using a separatory funnel at room temperature. The solvents were removed using a rotary evaporator (Buchi R210; Switzerland, Flawil) at 35°C under vacuum. From the hexane fraction (GNSbM-F-Hex), 0.1330 g was obtained. The dichloromethane fraction (GNSbM-F-DCM) yielded 2.3697 g, the ethyl acetate fraction (GNSbM-F-EtOAc) yielded 10.5817 g, and the aqueous fraction (GNSbM-F-Aq) yielded 16.8156 g.

The hexane fraction (GNSbM-F-Hex) was submitted to column chromatography, with an extract/silica ratio of 1 : 200. The column was eluted according to a gradient of increasing polarity, from DCM : MeOH : H_2_O (67 : 28 : 5) to DCM : MeOH : H_2_O (55 : 40 : 5), obtaining a total of 12 fractions of GNSbF-Hex (GNSbF-Hex-1–12). The GNSbF-Hex-3 fraction was purified by column chromatography, with an isocratic system of Hex : EtOAc (90 : 10) to get compound **1**. The spectral properties of this compound, including ^1^H-NMR and ^13^C-NMR data, were identical to those previously described in the literature for the lupeol. [9].

Lupeol (**1**) C_30_H_50_O crystals, m.p. 215–216°C. ^1^H-NMR (CDCl_3_, 400 MHz): *δ* (ppm); 4.68, 4.56 (2H, s, H-29a, 29b), 3.18 (1H, dd, *J* = 4.76 Hz, 11.2 Hz, H-3), 1.25, 1.02, 0.96, 0.94, 0.82, 0.78, 0.75, (each 3H, s, CH_3_ × 7). ^13^C-NMR (CDCl_3_, 100 MHz); *δ* (ppm); 38.2 (C-1), 25.3 (C-2), 79.2 (C-3), 38.7 (C-4), 55.4 (C-5), 18.5 (C-6), 34.4 (C-7), 40.9 (C-8), 50.6 (C-9), 37.3 (C-10), 21.1 (C-11), 27.6 (C-12), 39.0 (C-13), 42.9 (C-14) 27.6 (C-15), 35.7 (C-16), 42.9 (C-17), 48.5 (C-18), 48.1 (C-19), 151.1 (C-20), 29.9 (C-21), 40.2 (C-22), 28.1 (C-23), 15.5(C-24), 16.3 (C-25), 16.2 (C-26), 14.7 (C-27), 18.5 (C-28), 109.5 (C-29), 19.5 (C-30).

The ethyl acetate fraction (GNSbM-F-EtOAc) was purified by column chromatography, with an extract/silica ratio of 1 : 200. The column was eluted with DCM : MeOH : H_2_O 85 : 25 : 4 obtaining a total of 11 fractions GNSbMF-EtOAc (GNSbMF-EtOAc-1-11). The fraction GNSbMF-EtOAc-2 was also purified by column chromatography, with an isocratic system of EtOAc-MeOH 90 : 10 to get compound **2**, identified as 3′-O-methyl ellagic acid 4-O-*β*-D-rhamnopyranoside.

3′-O-Methyl ellagic acid 4-O-*β*-D-rhamnopyranoside (**2**) C_21_H_18_O_12_, white amorphous powder, m.p. 248–250°C. ^1^H-NMR (C_5_D_5_N, 400 MHz): *δ* (ppm) 8.58 (1H, s, H-5), 8.14 (1H, s, H-5′), 6.53 (1H, d, *J* = 1.5 Hz, H-1″), 4.98 (1H, br, H-2″), 4.88 (1H, d, *J* = 3.4 Hz, H-3″), 4.85 (1H, d, *J* = 3.4 Hz, H-5″), 4.51 (1H, t, *J* = 9.4 Hz, H-4″), 4.28 (3H, s, 3′-OCH_3_), 1.76 (3H, d, *J* = 6.2 Hz, CH_3_-6″); ^13^C NMR: (C_5_D_5_N, 100 MHz) *δ* (ppm): 113.3 (C-1), 113.3 (C-1′), 135.9 (C-2), 143.2 (C-2′), 142.7 (C-3), 139.5 (C-3′), 146.7 (C-4), 152.8 (C-4′), 111.3 (C-5), 110.7 (C-5′), 114.3 (C-6), 113.4 (C-6′), 158.8 (C-7), 158.0 (C-7′), 100.7 (C-1″), 70.0 (C-2″), 71.1 (C-3″), 72.8 (C-4″), 70.4 (C-5″), 17.2 (C-6″), 59.8 (3′-OMe).

The fraction GNSbMF-EtOAc-3 was purified by column chromatography, with an isocratic system of EtOAc : MeOH 85 : 15, to get compound **3**, identified as 19-*α*-hydroxy-asiatic acid monoglucoside.

19-*α*-Hydroxy-asiatic acid monoglucoside (**3**) C_36_H_58_O_11_, white amorphous powder, m.p. 220–221°C. ^1^H-NMR (DMSO-d6, 100 MHz): *δ* (ppm); 5.23 (1H, d, *J* = 7.9 Hz, H-1′), 4.29 (1H, s, H-2), *δ*; 4.15 (1H, m, H-5′); 4.28–4.57 (5H, m, H-2′, H-3′, H-4′, H-6′), 3.82 (1H, d, *J* = 9.3 Hz, H-3), 5.23 (1H, m, H-12) 3.12 (1H, d, *J* = 14.3 Hz, H-18), 1.27, 1.08, 0.94, 0.65, 0.53 (each 3H, s, CH_3_ × 5); ^13^C-NMR (DMSO-d6, 100 MHz); *δ* (ppm); 47.5 (C-1), 67.7 (C-2), 78.2 (C-3), 42.7 (C-4), 47.1 (C-5), 17.7 (C-6), 34.9 (C-7), 41.4 (C-8), 46.8 (C-9), 37.4 (C-10), 16.8 (C-11), 127.2 (C-12), 138.4 (C-13), 41.3 (C-14) 28.8 (C-15), 24.4 (C-16), 46.8 (C-17), 53.4 (C-18), 72.4 (C-19), 41.3 (C-20), 32.2 (C-21), 36.9 (C-22), 67.7 (C-23), 13.7 (C-24), 16.9 (C-25), 16.8 (C-26), 23.5 (C-27), 175.8 (C-28), 16.6 (C-29), 16.4 (C-30), 94.2 (C-1′), 74.2 (C-2′), 76.8 (C-3′), 71.8 (C-4′), 75.8 (C-5′), 69.7 (C-6′).

### 2.6. Cell Culture and Treatments

Two human colon carcinoma cell lines were used to analyze the antiproliferative effect of the *G. neuberthii* extracts: RKO and SW613-B3. Cells were grown at 37°C and 5% CO_2_ atmosphere, in RPMI medium supplemented with 10% FBS (Sigma Aldrich, USA), 0.1 mg/mL penicillin, 100 UI/mL streptomycin, and 2 mM L-glutamine (all reagents were from Gibco-Thermo Fisher Scientific, USA). Twenty-four hours after seeding, cells were treated for 48 h with the *G. neuberthii* extracts at 50 *μ*g/mL or increasing doses or 0.3 *μ*g/mL Doxorubicin (Dxo–Sigma Aldrich, USA; stock solution: 2 mg/mL in water). Final concentration of DMSO, <0.2% (v/v), in the culture medium did not alter the tested activities.

### 2.7. Viability Assay

Different *G. neuberthii* extracts were evaluated on cell proliferation by the MTS metabolic viability assay, according to Guamán-Ortiz et al. (2015). [10] Briefly, 2 × 10^3^ cells were seeded in 96-well plates in 100 *μ*L of medium per well and incubated for 24 h. Then, cells were treated in triplicate for 48 h either with 50 *μ*g/mL of each extract or 0.3 *μ*g/mL Dxo. Four hours before finishing the treatment, 20 *μ*L of Cell Titer 96 Aqueous One Solution cell proliferation reagent (Promega, USA) was added to each well. The plates were then maintained for 4 h at 37°C; the absorbance of each sample was measured with a microplate reader (Megallan, Tecan, Switzerland) at a wavelength of 492 nm. Absorbance from control was used as reference values (100% of viability) to normalize the data of treated samples. In order to calculate the IC_50_, the most active extracts were selected and exposed to the cells for 48 h with increasing concentrations (5–50 *μ*g/mL) and processed as above.

### 2.8. Morphological Analysis

Both cell lines were exposed to the most active extract of *G. neubertii* (GNSbM) in order to observe the induced effect. In brief, 5 × 10^4^ cells/mL were seeded in 3.5-cm diameter Petri dishes and incubated for 24 h. Next, cells were exposed to the extract for 48 h with their representative IC_50_ calculated. Cells were then observed using a light microscope (Axioskop 2 plus–Zeiss, Germany) equipped with a 40x objective. Images were acquired with a digital camera Basler scA1300-32 fm using its software.

### 2.9. Cell Cycle Analysis

Cell cycle distribution was evaluated using propidium iodide (PI, P4170, Sigma Aldrich, USA) staining processed according to the previous protocol, Bailon-Moscoso et al. [11]. In summary, cells were seeded in 6-well plates at a density of 1 × 10^6^ cells in 2 mL of medium per well and incubated for 24 h. Cells were then treated for 48 h with 20, 30, and 50 *μ*g/mL of GNSbM extract or 0.3 *μ*g/mL Dxo. Detached and attached cells were harvested and washed with PBS. Cell pellets were obtained and resuspended in 100 *μ*L of PBS, fixed with absolute ethanol, and maintained at −20°C for 24 h. Cells were washed with PBS and incubated in the dark for 30 min at room temperature in the staining buffer (50 *μ*g/mL PI, 0.1% sodium citrate, 0.1% Triton-X-100, and 100 *μ*g/mL RNase A). Cells in the *G*_1_, S, and *G*_2_/M-phase were subsequently analyzed using a FACSCanto II flow cytometer (Becton Dickinson, USA). Acquired data were analyzed using DIVA and ModFit LT software (Becton Dickinson).

### 2.10. Cloning Assay

To evaluate the clonogenic capacity, 2.5 × 10^2^ cells were seeded in duplicate in 6 cm diameter Petri dishes in 2 mL of medium and incubated for 24 h. Cells were then treated for 48 h with 20, 30, and 50 *μ*g/mL of GNSbM extract or 0.3 *μ*g/mL Dxo, washed with BPS, incubated with 2 mL of complete medium for 7 days, and then processed according to Guamán-Ortiz et al. [12]. Colony-forming ability data were expressed as a percentage relative to control.

### 2.11. Western Blot Analysis

To determine the induced cell death pathway, 20, 30, and 50 *μ*g/mL of GNSbM extract were exposed on both cell lines for 48 h. Additionally, as positive controls: cells were exposed for 10 min to UV radiation (Osram, G30T8, 30W Germicidal UV-C Lamp, 254 nm) for apoptosis induction [13] or for 1 h to PBS for starvation-induced autophagy [14], before being harvested. Apoptotic and autophagic proteins were analyzed through Western Blot analysis. The methodology was applied according to Bailon-Moscoso et al. [15]. Briefly, separated proteins from a SDS-PAGE were transferred to a PVDF membrane (IPVH00010, Immobilon-P, 0.45 *μ*m, EMD/Millipore, Billerica, Boston, MA, USA) and incubated with primary antibodies: p53 (sc-81168), Beclin-1 (sc-48341), SQSTM 1/p62 (sc-48402) (Santa Cruz Biotechnology, USA), PARP (#9542), Bax (#2774), Bcl-2 (#15071) LC3A/B (#12741), and *β*-Tubulin (#2128) (Cell Signaling Technology, USA), as indicated by the manufacturer for immunoblotting. Secondary antibodies, anti-mouse IgG, HRP-linked (#7076, Cell Signaling Technology, USA), and anti-rabbit IgG, HRP-linked (#7074, Cell Signaling Technology, USA), were subsequently used. Immunoreactive bands were visualized using an enhanced chemiluminescence Luminata™ Crescendo Western HRP Substrate or Luminata™ Forte Western HRP Substrate (Millipore-Merck, Germany).

### 2.12. Statistical Analysis

Statistical analyses were carried out in GraphPad Prism 4 (GraphPad Software, USA). All data were reported as the means ± SEM of three independently performed experiments, as detailed in each figure. The statistical significance was obtained with one-way analysis of variance (ANOVA) followed by the Dunnett posttest. A *P* < 0.05 was considered to be statistically significant comparing the control to the samples.

## 3. Results and Discussion

### 3.1. Preliminary Phytochemical Study of Extracts

The phytochemical screening tests on extracts revealed the presence or absence of the main secondary metabolites and other phytochemicals based on the presence or absence of expected color changes (Table 1). The *G. neuberthii* fruit extracts in methanol (GNFM) are richer in secondary metabolites; the methanolic extracts of stem bark (GNSbM) contained alkaloids, flavonoids, tannins, quinones, and saponins. The highest percentage of yield was obtained from the methanol fraction of leaves GNLM (22.97%), followed by that of hexane fraction of fruit GNFH (19.6%), and methanol fraction of stem bark GNSbM (9.4%). The lowest percentage of yield was obtained from the hexane fraction of seed GNSH (0.17%). There was also variation in the physical appearance of the extracts (Supplementary Material, S1).

### 3.2. Cytotoxic Effect on Human Tumor Cell Lines

In order to analyze the antiproliferative effect of *G. neuberthi* on colon cancer cells, all the extracts obtained were then evaluated.  Table 2 describes the viability percentages using 50 *μ*g/mL of the different *G. neuberthii* extracts or Dxo 0.3 *μ*g/mL on the human tumor cell lines after 48 h of treatment. Doxorubicin, an anthracycline antibiotic, has proved to induce cytotoxicity, cell cycle arrest, and apoptosis in a wide variety of tumor cell lines, including colon cancer cells; however, its clinical use is limited due its cardiotoxicity effect [16–18]. Cell growth viability was measured using the MTS assay, considering the control as 100% of viability. As expected, the SW613-B3 cell line (with p53 mutated) was more resistant to the treatment, in contrast to the RKO cell line. The extract GNSbM, whose extracting method was by maceration in methanol, demonstrated to be the most cytotoxic, with values under 20% of viability on both wt p53 RKO and SW613-B3 with mutated p53.

**Figure 1:**
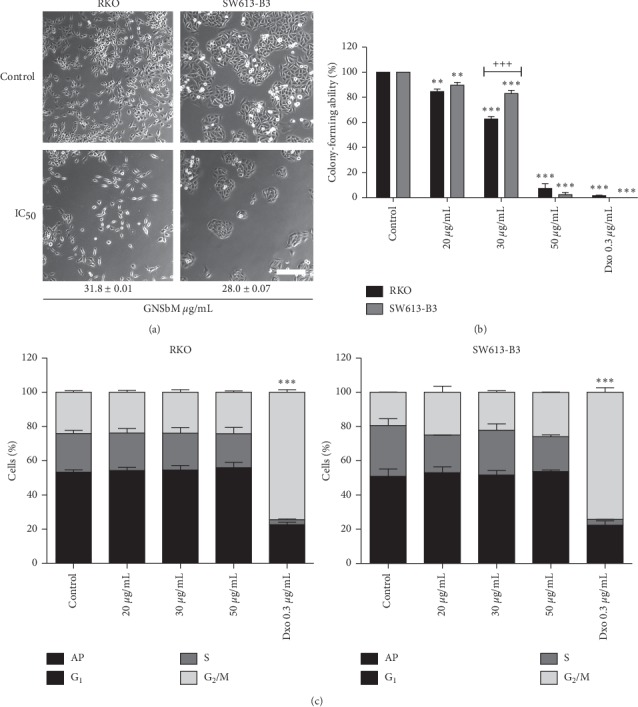
GNSbM extract demonstrated to be cytotoxic on RKO and SW613-B3 cell lines. (a) Morphological changes induced after 48 h of exposure at IC_50_ doses of the extract. Scale bar: 50 *μ*m. (b) Cells were exposed to the extract for 48 h and formed colonies were counted based on clonogenic survival assay seven days after the treatment. The number of counted colonies was expressed as a percentage relative to control (defined as 100%). Data represented the mean ± SEM (*n* = 6) of three independent experiments. The tests for significance were limited to ANOVA-Dunnet posttest, ^*∗∗*^*P* < 0.001, ^*∗∗∗*^*P* < 0.0001 vs. control; ^+++^*P* < 0.001 RKO vs. SW613-B3 at IC_50_ dose. (c) Cells were exposed to the extract for 48. (h) Percentage of cells in the *G*_1_, S, and *G*_2_/M-phases of the cell cycle was analyzed using a Facscanto II flow cytometer. Data were acquired and analyzed using DIVA software (Becton Dickinson). Data represented the mean ± SEM (*n* = 6) of three independent experiments. The tests for significance were limited to ANOVA-Dunnet posttest, ^*∗∗∗*^*P* < 0.0001 for *G*_1_, S, and *G*_2_/M.

According to these results, the IC_50_ was calculated from the GNSbM extract (Figure 1(a)), which was 28 and 31.8 *μ*g/mL in SW613-B3 and RKO, respectively. As observed, after exposing to the IC_50_ of GNSbM, that is approximately the same, both cell lines decrease in cell population, suggesting therefore that the effect observed is not dependent on p53 status.

The marked difference between these cell lines reveals that the SW613-B3 cells have the potential for recovery after treatment because of the results observed in colony-forming ability assay, where the cells were exposed to three different doses of GNSbM, 20, 30, and 50 *μ*g/mL. As observed in Figure 1(b), colony-forming ability decreases in a dose-dependent manner in both cell lines; however, the SW613-B3 cell line was less affected after 20 *μ*g/mL (90%) and IC_50_ (80%), in contrast to the RKO cell line, which was more sensitive (60%). To note, SW613-B3 cell line has demonstrated to be resistant to different treatments. [12] Nevertheless, colony-forming ability decreases dramatically in both cell lines at the higher concentration (50 *μ*g/mL). On the other hand, p53 has a well-known role in cell cycle progression, which after transactivation induces cell cycle arrest [19, 20]; however, the statistical analysis of cell cycle distribution revealed that no significant cell cycle changes were occurring after the treatment of both wt and mutated p53 cell lines, as observed in those treated with Dxo (Figure 1(c)).

### 3.3. No Apoptosis Was Detected after Treatment with GNSbM

**Figure 2:**
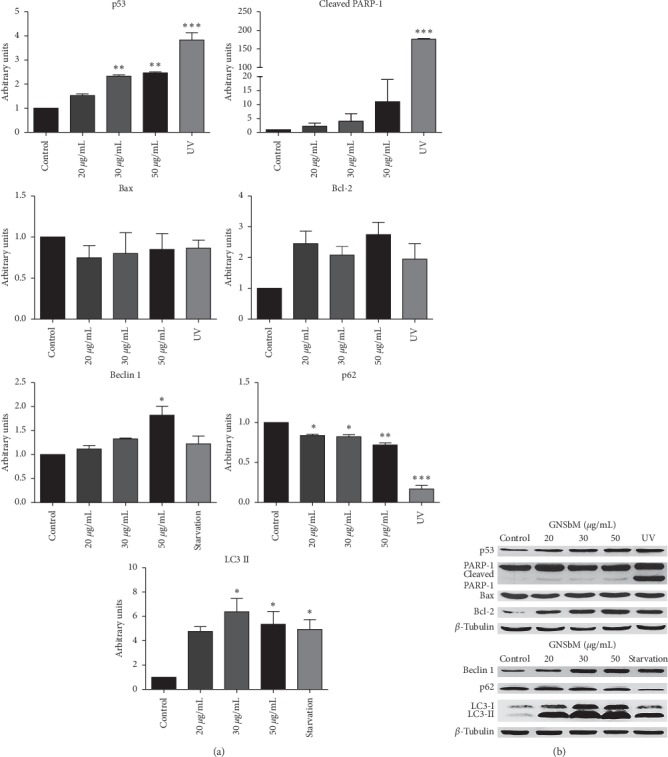
GNSbM extract induces autophagy on RKO line cell. Cells were exposed to GNSbM or UV radiation or starvation in PBS. Total protein was separated in a SDS-PAGE followed by Western blot analysis with indicated antibodies against p53, apoptotic and autophagy biomarkers, and tubulin as a loading control. (a) Quantification of the level of p53 expression and the apoptotic and autophagy biomarkers. Data represented the mean ± SEM (*n* = 6) of three independent experiments. The tests for significance were limited to ANOVA-Dunnet posttest: ^*∗*^*P* < 0.01, ^*∗∗*^*P* < 0.001, ^*∗∗∗*^*P* < 0.0001 vs. control. (b) Western blot pictures demonstrating the effect observed in RKO cell line. Although p53 showed to be overexpressed in a dose-dependent manner, no apoptotic activity was detected, and autophagy activity was monitored after treatment.

To further explore the type of cell death induced by the plant extract, both apoptosis and autophagy pathways were analyzed by Western blotting. It is also well known that p53 is involved in multiple cell death pathways, such as apoptosis and autophagy. [21, 22] In the apoptotic pathway, p53 is upregulated to transactivate and phosphorylate the Bax protein, thereby inducing the activation of the intrinsic apoptosis; at the same time Bcl-2, an antiapoptotic protein, is downregulated in the presence of p53. [22] As observed in Figures 2(a) and 2(b), no overexpression of Bax protein was detected in the RKO cell line, despite the overexpression of p53 in a dose-dependent manner; furthermore, an increase of Bcl-2 expression was detected in the Western blot assay (Figure 2(b)), although this did not reach statistical significance relative to the control (Figure 2(a)).

**Figure 3:**
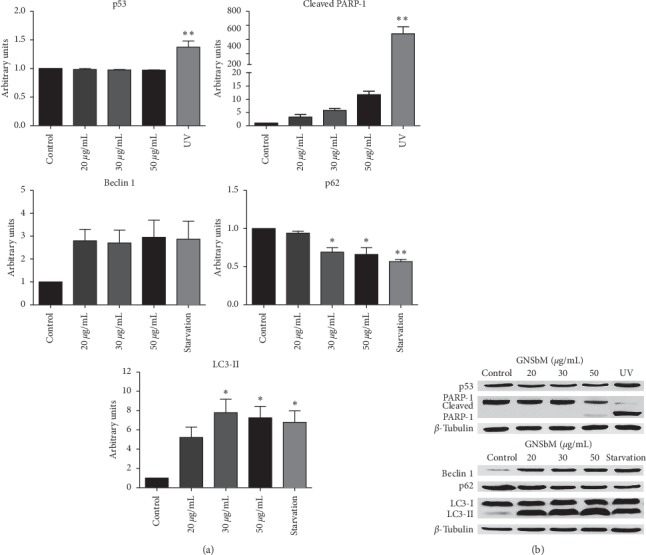
GNSbM extract induces autophagy on SW613-B3 cell line. Cells were exposed to GNSbM or UV radiation or starvation in PBS. Total protein was separated in a SDS-PAGE followed by Western blot analysis with indicated antibodies against p53, apoptotic and autophagy biomarkers, and tubulin as a loading control. (a) Quantification of the level of p53 expression and the apoptotic and autophagy biomarkers. Data represented the mean ± SEM (*n* = 6) of three independent experiments. The tests for significance were limited to the ANOVA-Dunnet posttest: ^*∗*^*P* < 0.01, ^*∗∗*^*P* < 0.001, ^*∗∗∗*^*P* < 0.0001 vs. control. (b) Western blot pictures demonstrating the effect observed in Sw613-B3 cell line. No p53 expression was observed as expected although autophagy activity was detected after treatment.

Additionally, in the SW613-B3 cell line, the upregulation of p53 was not observed in any of the GNSbM doses used as expected (Figures 3(a) and 3(b)). In both cell lines, no proteolysis of PARP-1 protein, an apoptotic marker [23, 24], was detected after the GNSbM treatments (Figures 2 and 3), in contrast with the cleavage visible in cells exposed to UV radiation. [25].

### 3.4. GNSbM Extract Induces Autophagy

Autophagy pathway is activated after starvation conditions [26, 27], which induces the formation of autophagic vesicles or vacuoles called autophagosomes at the level of the cytoplasm. Therefore, the presence of vacuoles in both cell lines, observed in morphological analysis (Figure 1(a)), suggested the possible activation of the autophagic pathway [12, 28]; thus, biomarkers for autophagy were evaluated. In this pathway, Bcl-2 is associated with Beclin-1. Once Beclin-1 is released, the autophagic pathway is activated [29]; LC3-I is converted into its active form LC3-II to induce the phagophore formation in the nucleation phase to enclose the obsolete proteins and organelles, tagged with p62, for degradation [30–33]. Forced autophagy could generate severe damage that ends in the death of the cell [27]. In Figures 2(b) and 3(b), an upregulation of Beclin-1 was observed in both cell lines in a dose-dependent manner, although these results did not reach statistical significance. Dissociation between Bcl-2 and Beclin-1 is necessary for the initiation of autophagy; therefore, the increase of Bcl-2 observed in the RKO cell line (Figure 2(b)) could be explained by this dissociation. Likewise, an increase in LC-3II was detected in both the RKO and SW613-B3 cell lines in a dose-dependent manner. The final step in autophagy is the degradation of charges in which p62 is involved (being also degraded). [32] As observed in both cell lines, p62 results in a decrease (Figures 2(a) and 3(a)), therefore elucidating the termination of the autophagic process and cell death pathway. Remarkably, it is possible that autophagy is playing a double role in the SW613-B3 cell line. It is well known that the autophagy mechanism is also active in tumor cell lines and has a role in survival [22, 34]; as observed in the clonogenic assay, the SW613-B3 cell line is able to recover after treatment at the IC_50_ dose (Figure 1(b)).

### 3.5. Isolation and Identification of Secondary Metabolites Isolated from GNSbM

**Figure 4:**
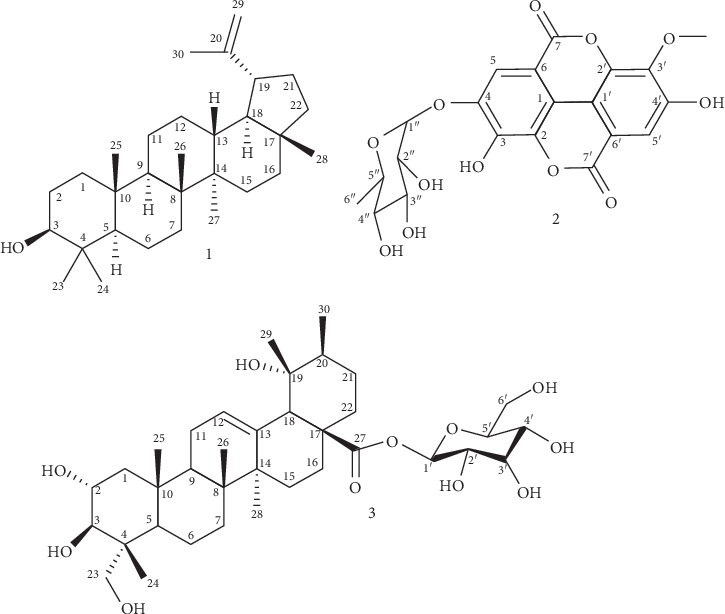
Structures of compounds isolated from methanolic extract from the stem bark of *G. neuberthii* (GNSbM).

Phytochemical investigation of methanolic extract from the stem bark of *G. neuberthii* (GNSbM) led to the isolation and identification of three compounds identified as: lupeol (**1**), 3′-O-methyl ellagic acid 4-O-*β*-D-rhamnopyranoside (**2**), and 19-*α*-hydroxy-asiatic acid monoglucoside (**3**), whose structures are shown in Figure 4. The spectral properties of these known compounds, including the ^1^H NMR and ^13^C NMR data, were identical to those previously described in the literature [9].

Compound **1** was crystals. Its ^1^H NMR spectra in CDCl_3_ showed six singlets, corresponding to tertiary methyl groups, between 0.75 and 1.02 ppm and a singlet at 1.25 ppm, typical of a methyl group in an isopropenyl system. Two olefinic protons at 4.68 and 4.56 ppm are consistent with the methylene group of the same propylenic system. The ^13^ C NMR data showed the characteristic signals of C-3 at 79.2 ppm, C-20 at 151.1 ppm, and C-29 at 109.5 ppm. [9] Compound **2** was a white amorphous powder. Its ^1^H NMR spectra in deuterated pyridine showed two singlet signals at *δ* 8.58 and 8.14. A broad signal at 5.31 ppm suggested a structure with some hydroxyls groups, which was corroborated with the few multiplets signals typical for carbohydrate moieties between 4.53 and 5.04 ppm. Among these signals, one singlet at *δ* 4.28 integrating for 3H suggested a methoxy group in this compound. The broad band carbon NMR spectra showed 21 signals including 11 quaternary carbons indicating a very conjugated aromatic structure. From these signals, two were assigned to *α*, *β*-unsaturated lactone carbonyls carbons at 158.0 and 158.2 ppm. The signals corresponding to the carbohydrate, analyzed together with the proton spectra, indicated that it was the *β*-D-glucopiranoside. All the data obtained by NMR were ascribable to a derivative of ellagic acid. The comparison of physical and spectroscopic data [35] indicated that the isolated compound **2** was 3′-O-methyl ellagic acid 4-O-*β*-D-glucopyranoside. Compound **3** was a white amorphous powder. The NMR spectra were taken in deuterated DMSO, submitted to analyses. The ^1^H NMR showed the resonances of five quaternary methyl groups at 0.52, 0.54, 0.94, 1.08, and 1.26 ppm, together with a methyl doublet at *δ* 0.86. An olefinic proton in 5.14 ppm suggested an ursane-type triterpene for the aglycone of this glycosylated compound. Few multiplets between 3.8 and 4.60 ppm were ascribable to a sugar moiety. The carbon NMR spectra analyzed with the help of DEPT experiment revealed for the aglycone triterpene 30 carbons, including six methyl groups, nine methylene groups, counting one oxygenated, seven methine groups with two oxygenated at 77.7 and 67.7 ppm, and one olefinic group at 138.4 ppm; eight quaternary carbons comprising the olefinic at 127.2 ppm, one oxygenated at 72.4 ppm, and the carboxyl ester group at 175.8 ppm. Comparative study of the resonances of the sugar indicated that it was the *β*-D-glucoside. The 2D experiments, COSY, HMQC, and HMBC were in agreement with the proposed structure. All the data were compared with the literature [36, 37] to finally consider this compound as 19-hydroxy-asiatic acid monoglucoside.

Regarding this, lupeol (**1**), 3′-O-methyl ellagic acid 4-O-*β*-D-rhamnopyranoside (**2**), and asiatic acid-*β*-D-glucoside (**3**) are reported here for the first time in this genus. For instance, lupeol has been widely reported for its anticancer effect against various cancer cells, such as oral cancer, pancreatic cancer, gallbladder cancer, prostate cancer, and colorectal cancer. [38–41] Also, asiatic acid is known to be cytotoxic to several tumor cell lines. However, asiatic acid-induced cell death was mainly apoptotic, demonstrated in colon cancer RKO cells [42, 43]. Hence, the presence of these phytochemicals in GNSbM might be synergistically responsible for the autophagy-inducing effect, as suggested by our results.

## 4. Conclusions

In summary, different extracts of *G. neuberthii* were evaluated; the one with the greatest cytotoxic effect was the so-called GNSbM, which considerably reduced cell viability in both RKO cell line, with wt p53, and SW613-B3, with mutated p53, in a dose-dependent manner after 48 h of exposure. In addition, activation of the apoptotic route was discarded and the evidence of autophagic activity was detected. Finally, three compounds were identified in this extract: lupeol, 3′-O-methyl ellagic acid 4-O-*β*-D-rhamnopyranoside, and asiatic acid-*β*-D-glucoside, which have been shown to have antitumor effects.

## Figures and Tables

**Table 1:** Phytochemical constituents of extract from the aerial parts of *G. neuberthi*.

Test	Fruit			Seed			Stem bark			Leaves		
	Hex GNFH	EtOAc GNFEa	MeOH GNFM	Hex GNSH	EtOAc GNSEa	MeOH GNSH	Hex GNSbH	EtOAc GNSbEa	MeOH GNSbM	Hex GNLH	EtOAc GNLEa	MeOH GNLM
Proteins	−	−	+	−	+	−	−	−	−	−	−	−
Carbohydrates	−	−	+	−	−	−	−	−	−	−	−	+
Fats	+++	++	+	++	−	++	++	+	−	+	−	−
Alkaloids	++	++	++	−	−	−	−	+	+	++	−	+
Terpenoids, steroids	−	−	+	−	−	−	−	−	−	−	−	−
Flavonoids	+	+	+	−	+	−	−	−	+	+	+	+
Saponnins	−	−	+	−	−	−	−	+	+++	−	−	+
Quinones	−	−	++	−	−	−	−	−	++	−	−	++
Tannins	−	−	+	+	−	+	−	+	+	−	+	+

Hex = hexane extract, EtOAc = ethyl acetate extract, MeOH = methanol extract, +++ = very strong positive, ++ = strong positive, + = fair positive, − = absent.

**Table 2:** Cell Growth Viability: Cell lines were treated for 48 h with 50 *μ*g/mL of *G. neuberthii* extract.

Part of the plant	Extract	RKO	SW613-B3
Fruit	GNFH	96 ± 6.79	NE
	GNFEa	NE	NE
	GNFM	72.2 ± 6.38	NE

Seed	GNSEa	81.0 ± 7.78	NE
	GNSM	74.7 ± 4.36	95.0 ± 5.31

Stem bark	GNSbEa	NE	NE
	GNSbM	15.3 ± 3.61	10.4 ± 2.24

Leaves	GNLM	91.5 ± 4.95	NE

Control	0.3 *μ*g/mL Dxo	10.8 ± 4.02	48.2 ± 5.62

Control was considered as 100% of cell viability; three independent experiments in triplicate were performed. NE = no effect.

## Data Availability

The datasets generated and/or analyzed during the current study are available from the corresponding author on reasonable request.

## Supplementary Materials

Supplementary Materials Different parts of the G. *neubetthii* were macerated for 72 h in a light‐free environment with three dissolvents separately: hexano, ethyl acetate, and methanol. The extracts were concentrated at 50 mbar and 37° C on a rotary evaporator. The appearance and weight of the extracts were recorded, and the yield was calculated.
