# Supplementary material for: Cytotoxic Property of Grias neuberthii Extract on Human Colon Cancer Cells: A Crucial Role of Autophagy
Source: Evid Based Complement Alternat Med. 2020 Apr 1;2020:1565306. doi: 10.1155/2020/1565306 (PMC7152961; doi:10.1155/2020/1565306)
Supplement: Supplementary Materials — Different parts of the G. neubetthii were macerated for 72 h in a light‐free environment with three dissolvents separately: hexano, ethyl acetate, and methanol. The extracts were concentrated at 50 mbar and 37° C on a rotary evaporator. The appearance and weight of the extracts were recorded, and the yield was calculated. [file 1565306.f1.docx]

**Supplementary material**

Cytotoxic property of *Grias neuberthii* extract on human colon cancer cells: A crucial role of autophagy

Luis Miguel Guamán-Ortiz,^[a]^ Juan Carlos Romero-Benavides,^[b]^ Alirica I. Suarez,^[b,c]^ Stephania Torres-Aguilar,^[a]^ Paola Castillo-Veintimilla,^[b,d]^ Jimmy Samaniego-Romero,^[a]^ Kevin Ortiz-Diaz, ^[a]^ Natalia Bailón-Moscoso,*^[a]^

[a] L. M. Guamán Ortiz, Stephania Torres-Aguilar, Jimmy Samaniego-Romero, Kevin Ortiz-Diaz, Natalia Bailón-Moscoso

Departamento de Ciencias de la Salud

Universidad Técnica Particular de Loja

San Cayetano Alto, Calle París, 1101608 Loja (Ecuador)

E-mail: ncbailon@utpl.edu.ec

[b] J. C. Romero-Benavides, A. I. Suárez, P. Castillo-Veintimilla

Departamento de Química y Ciencias Exactas

Universidad Técnica Particular de Loja

San Cayetano Alto, Calle París, 1101608 Loja (Ecuador)

[c] A. I. Suárez

Facultad de Farmacia

Universidad Central de Venezuela

Ciudad Universitaria de Caracas, Los Chaguaramos 1050 Caracas (Venezuela)

[d] P. Castillo-Veintimilla

Programa Nacional para el Abordaje Multidisciplinario de las parasitosis desatendidas en el Ecuador PROPAD, Instituto Nacional de Investigaciones en Salud Pública LIP Av. Julián Coronel 905 entre Esmeraldas y José Mascote, Guayaquil (Ecuador)

**S1**. Yield and physical appearance of extracts of *Grias neuberthi*

| ***Morphological structure used*** | ***Extract*** | ***Code*** | ***Physical appearance*** | ***Weight (g)*** | ***Yield (%)*** |
| --- | --- | --- | --- | --- | --- |
| Fruit | Hexane | GNFH | Oily liquid, orange | 61.83 | 19.60 |
|  | Ethyl acetate | GNFEa | Oily liquid, orange | 8.53 | 2.70 |
|  | Methanol | GNFM | Semi solid (gumy), brown | 14.00 | 4.44 |
| Seed | Hexane | GNSH | Oily liquid, orange | 0.72 | 0.17 |
|  | Ethyl acetate | GNSEa | Oily liquid, yellowish green | 5.83 | 1.37 |
|  | Methanol | GNSM | Semi solid (gumy), brown | 24.73 | 5.82 |
| Stem bark | Hexane | GNSbH | Yelly, yellow | 0.49 | 0.54 |
|  | Ethyl acetate | GNSbEa | Solid, green | 0.21 | 0.23 |
|  | Methanol | GNSbM | Semi solid (gumy), brown | 8.47 | 9.40 |
| Leaves | Hexane | GNLH | Yelly, green | 0.28 | 3.40 |
|  | Ethyl acetate | GNLEa | Solid, green | 0.11 | 1.42 |
|  | Methanol | GNLM | Semi solid (gumy), brown | 2.85 | 22.97 |

Different parts of the *G. neubetthii* were macerated for 72 h in a light-free environmental whit three dissolvent separately: Hexano, Ethyl acetate, and Methanol. The extracts were concentrated at 50 mbar and 37°C on a rotary evaporator. The appearance and weight of the extracts were recorded and the yield was calculated.
